# Supplementary material for: Congenital Malformations in Sea Turtles: Puzzling Interplay between Genes and Environment
Source: Animals (Basel). 2021 Feb 8;11(2):444. doi: 10.3390/ani11020444 (PMC7915190; doi:10.3390/ani11020444)
Supplement: Supplementary file 1 [file animals-11-00444-s001.pdf]

Supplementary file

Playa Espíritu (FONATUR) is a protected area located in the northwest region, in the state of Sinaloa, Mexico (Figure S1A), where nests are relocated to protect them from extreme temperatures and predators (Figure S1B,C). Temperature is monitored and controlled by temperature sensors (Onset type, HOBO Pendant® UA-001-08 positioned at 30 cm depth into the sand) and shade structures; humidity is managed by a sand sanitisation procedure involving sand screening, aeration, flattening, and water sprinkling (Figure S2) resulting in consistent survival and hatching rates above 85% (Table S1).

Table S1. Temperature variation, hatching success, and malformed embryos or hatchlings (2016–2020) at Playa Espíritu, Sinaloa, Mexico.

Year	Temperature (°C) Min—Mean—Max	Hatching (%)	Number of Relocated Nests/Hatchlings	Number of Malformed Embryos or Hatchlings
2016	23.6—29.9—33.0	85	1027/69552	3
2017	24.1—29.7—34.2	87	1304/106419	2
2018	21.8—28.9—34.4	88	1049/82039	4
2019	24.6—30.3—34.0	89	656/55925	2
2020	26.4—30.7—34.2	87	221/16139	2

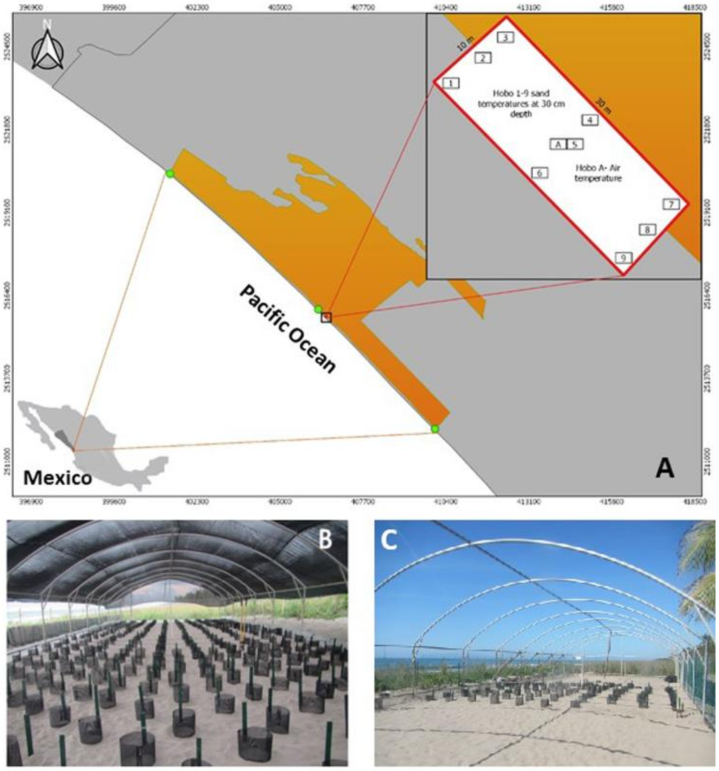

Figure S1. (**A**) Location of the turtle camp "Playa Espíritu" in Sinaloa, northwest Mexico; green dots on the map indicate temperature monitoring sites along the 12 Km beach, and rectangles (schematically represented at the top right corner) indicate position of HOBOs inside the hatchery, HOBOs 1-9 monitor sand temperature at 30 cm depth; HOBO A monitors air temperature. (**B**) and (**C**) Nest relocation and arrangement with (**B**) or without (**C**) a shade structure, depending on the temperature. This turtle camp has been operating since 2012

following a UNAM-FONATUR agreement authorised by the Ministry of the Environment (permit number SGPA/DGIRA/DG/1167/11), under the leadership of Raquel Briseño-Dueñas.

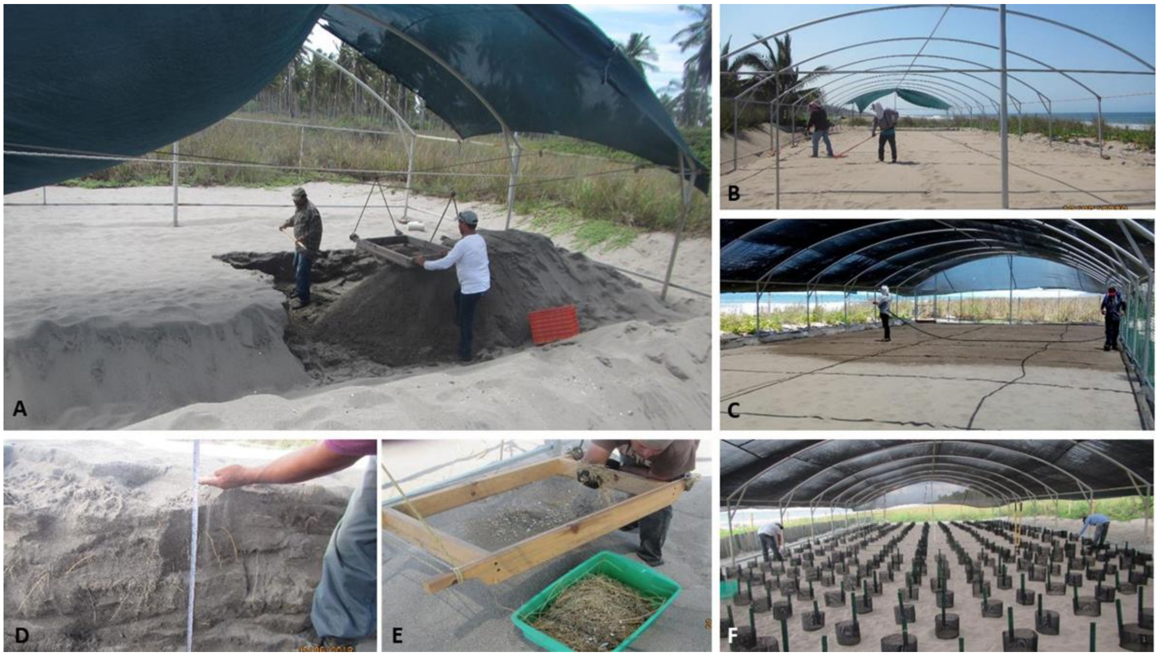

Figure S2. Different activities are carried out in the sea turtle camp Playa Espíritu (Sinaloa, Mexico) for the sand sanitization procedure during May to June, preceding the nesting season. The procedure includes sand screening and aeration to allow oxygenation (**A**); sand flattening (**B**); water sprinkling to control moist, and shade structure to control temperature (**C**); sand cleaning to 60 cm depth and exposure to sunlight (**D**); screening through a 0.6 cm mesh to control roots, grain size, and organic matter (**E**); nest allocation (**F**).
